# Diagnosis of gestational diabetes mellitus: falling through the net

**DOI:** 10.1007/s00125-015-3647-z

**Published:** 2015-06-14

**Authors:** Claire L. Meek, Hannah B. Lewis, Charlotte Patient, Helen R. Murphy, David Simmons

**Affiliations:** The Wellcome Trust–MRC Institute of Metabolic Science, Metabolic Research Laboratories, University of Cambridge, Addenbrooke’s Hospital, Box 289, Hills Road, Cambridge, CB2 0QQ UK; Wolfson Diabetes and Endocrinology Clinic, Cambridge University Hospitals, Addenbrooke’s Hospital, Cambridge, UK; Department of Clinical Biochemistry, Cambridge University Hospitals, Addenbrooke’s Hospital, Cambridge, UK; Department of Obstetrics and Gynaecology, Cambridge University Hospitals, The Rosie Hospital, Cambridge, UK; School of Medicine, University of Western Sydney, Campbelltown, Sydney, NSW Australia

**Keywords:** Diabetes, Diagnosis, Gestational, Macrosomia, Pregnancy, Screening

## Abstract

**Aims/hypothesis:**

Gestational diabetes mellitus (GDM) is associated with increased risks to mother and child, but globally agreed diagnostic criteria remain elusive. Identification of women with GDM is important, as treatment reduces adverse outcomes such as perinatal death, shoulder dystocia and neonatal hypoglycaemia. Recently, the UK’s National Institute for Health and Care Excellence (NICE) recommended new diagnostic thresholds for GDM which are different from the International Association of the Diabetes and Pregnancy Study Groups (IADPSG) criteria endorsed by the WHO. The study aim was to assess neonatal and obstetric outcomes among women who would test positive for the IADPSG criteria but negative for the NICE 2015 criteria.

**Methods:**

Data from 25,543 consecutive singleton live births (2004–2008) were obtained retrospectively from hospital records. Women were screened with a random plasma glucose (RPG; 12–16 weeks) and a 50 g glucose challenge test (GCT; 26–28 weeks). If RPG >7.0 mmol/l, GCT >7.7 mmol/l or symptoms were present, a 75 g OGTT was offered (*n* = 3,848).

**Results:**

In this study, GDM prevalence was 4.13% (NICE 2015) and 4.62% (IADPSG). Women who ‘fell through the net’, testing NICE-negative but IADPSG-positive (*n* = 387), had a higher risk of having a large-for-gestational-age (LGA) infant (birthweight >90th percentile for gestational age; adjusted OR [95% CI] 3.12 [2.44, 3.98]), Caesarean delivery (1.44 [1.15, 1.81]) and polyhydramnios (6.90 [3.94, 12.08]) compared with women with negative screening results and no OGTT (*n* = 21,695). LGA risk was highest among women with fasting plasma glucose 5.1–5.5 mmol/l (*n* = 167): the mean birthweight was 350 g above that of the reference population and 37.7% of infants were LGA.

**Conclusions/interpretation:**

The IADPSG criteria identify women at substantial risk of complications who would not be identified by the NICE 2015 criteria.

## Introduction

Gestational diabetes mellitus (GDM), defined as carbohydrate intolerance causing hyperglycaemia with first onset or recognition in pregnancy [1, 2], is increasing in incidence in many populations worldwide as obesity becomes more prevalent [3]. Untreated GDM results in poor maternal and fetal outcomes: women with GDM are more likely to suffer pre-eclampsia, operative delivery and stillbirth [4], and infants are at higher risk of preterm delivery and macrosomia or large for gestational age (LGA), which is associated with birth injury, respiratory distress and neonatal hypoglycaemia [5]. In the longer term, children born to mothers with GDM are at greater risk of obesity and type 2 diabetes in later life, a phenomenon attributed to the effects of intrauterine exposure to hyperglycaemia [6, 7].

Fortunately, many of these risks can be reduced by identification of GDM pregnancies and prompt intervention to reduce maternal antenatal hyperglycaemia [8, 9]. One barrier to case identification has been the lack of a universally accepted set of diagnostic criteria for GDM. The International Association of the Diabetes and Pregnancy Study Groups (IADPSG) proposed diagnostic criteria which were based upon an OR of 1.75 for negative pregnancy outcomes (Table 1) using data from the Hyperglycaemia and Adverse Pregnancy Outcomes (HAPO) study (75 g OGTT 0 h ≥5.1 mmol/l, 1 h ≥10.0 mmol/l, 2 h ≥8.5 mmol/l) [10, 11]. However, these criteria used lower fasting plasma glucose (FPG) thresholds than other criteria in common use (Table 1) and added a 1 h criterion, leading to concerns about increased diagnosis rates, resource allocation and increased medicalisation of pregnancy [12, 13]. The IADPSG criteria have been adopted by the WHO [2] and the ADA [14] but were not endorsed at the National Institutes of Health summit in the USA [12] nor by the National Institute for Health and Care Excellence (NICE) in the UK due to concerns about treatment costs and the limited evidence of benefit for treating at lower diagnostic thresholds. NICE proposed alternative criteria for adoption in 2015 (75 g OGTT 0 h ≥5.6 mmol/l; 2 h ≥7.8 mmol/l) [15].

**Table 1:** Current and recent criteria used for diagnosis of GDM based on the OGTT

OGTT criterion	IADPSG, WHO 2013 and ADA 2014 [11]	WHO 1999	Modified WHO 1999	Proposed NICE 2015	ACOG
Diagnostic requirements	One abnormality on 75 g OGTT	One abnormality on 75 g OGTT	One abnormality on 75 g OGTT	One abnormality on 75 g OGTT	Two abnormalities on 100 g OGTT
FPG, mmol/l (mg/dl)	≥5.1 (≥92)	≥7.1 (≥128)	≥6.1 (≥110)	≥5.6 (≥101)	≥5.3 (≥95)
OGTT 1 h glucose, mmol/l (mg/dl)	≥10.0 (≥180)	–	–	–	≥10.0 (≥180)
OGTT 2 h glucose, mmol/l (mg/dl)	≥8.5 (≥153)	≥7.8 (≥140)	≥7.8 (≥140)	≥7.8 (≥140)	≥8.6 (≥154)
OGTT 3 h glucose, mmol/l (mg/dl)	–	–	–	–	≥7.8 (≥140)

The aim of the current study was to retrospectively compare outcomes among women who would test positive for the IADPSG criteria for GDM but negative for NICE 2015 diagnostic criteria.

## Methods

### Population and standard care

Data from all singleton pregnancies (2004–2008) at Cambridge University Hospitals National Health Service Foundation Trust were obtained retrospectively from hospital medical and obstetric records as part of an approved service evaluation. At that time in our institution all pregnant women were invited to be screened at antenatal booking with a random plasma glucose (RPG; *n* = 17,736; typically at 12–16 weeks’ gestation). Women with RPG >7.0 mmol/l or a previous diagnosis of GDM were offered a 75 g OGTT. All women without known GDM/pre-existing diabetes were screened at 26–28 weeks with a 50 g glucose challenge test (GCT). Women with a GCT result >7.7 mmol/l were then referred for a 75 g OGTT [16]. Additional OGTTs were performed in later pregnancy if symptoms were present. Therefore, all women who had an OGTT (*n* = 3,848) had already had at least one abnormal result on glucose testing during pregnancy, symptoms consistent with hyperglycaemia, or GDM in a previous pregnancy. Women with known pre-existing diabetes were excluded from the study. The WHO 1999 criteria were used for GDM diagnosis until August 2007 (75 g OGTT 0 h ≥7.1 mmol/l; 2 h ≥7.8 mmol/l) and the modified WHO 1999 criteria thereafter (75 g OGTT 0 h ≥6.1 mmol/l; 2 h ≥7.8 mmol/l; Table 1). Following diagnosis, women with GDM were seen every 2–4 weeks at a multidisciplinary clinic, encouraged to monitor their blood glucose levels and offered lifestyle counselling. Women who had evidence of persistent hyperglycaemia were offered treatment with insulin, metformin or both [17]. Women with GDM were offered regular ultrasound scans during pregnancy (at 12, 20, 28 and 36 weeks), whereas non-diabetic women usually have two routine ultrasound scans (at 12 and 20 weeks).

### Laboratory analysis

Both venous and capillary samples were used during 2004 and 2008 for glucose testing in our institution. Venous blood was collected using fluoride oxalate tubes and analysed using a hexokinase method (Dimension RxL Max Clinical Chemistry System; Siemens Healthcare Diagnostics, Deerfield, IL, USA) in our laboratory accredited by Clinical Pathology Accreditation, UK. Capillary samples were analysed using the Bayer Elite glucose monitoring system (Bayer, Newbury, UK). Although both laboratory and point-of-care methods were regularly calibrated, small differences exist between capillary and venous glucose testing [18]. The same diagnostic criteria were used for both capillary and venous tests.

### Definitions

Macrosomia was defined as birthweight >4 kg. LGA was defined as birthweight >90th percentile for gestational age and was calculated for babies born at 24–41 weeks’ gestation using the WHO weight percentile calculator with a mean birthweight of 3,542 g (SD 437 g) (World Health Organisation Weight percentiles calculator, available from www.who.int/reproductivehealth/topics/best_practices/weight_percentiles_calculator.xls; accessed 20 April 2015) [19–21]. Pre-eclampsia was defined as systolic blood pressure ≥140 mmHg and/or diastolic blood pressure ≥90 mmHg on two or more occasions with proteinuria ≥1+ on dipstick. Patients with no blood pressure recorded who were on the pre-eclampsia treatment pathway before or during labour were also considered to have pre-eclampsia. Patients with chronic hypertension prior to pregnancy were not considered to have pre-eclampsia. Preterm delivery was defined as delivery prior to 37 weeks’ gestation. Polyhydramnios was defined as excessive amniotic fluid, corresponding to the deepest vertical pool ≥8 cm on ultrasound or an amniotic fluid index >95th percentile for the corresponding gestational age. Antepartum haemorrhage was defined as any blood loss from the vagina after the 24th week of gestation. Postpartum haemorrhage was defined as blood loss of >500 ml following delivery, or the requirement for a blood transfusion.

### Statistical analysis

**Fig. 1:**
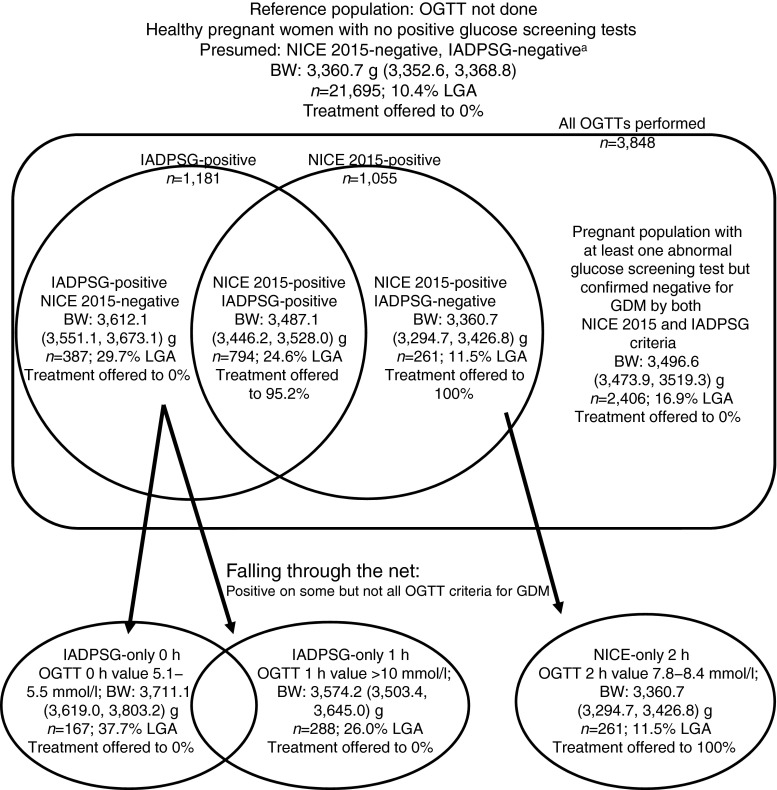
Classification of pregnant women according to GDM diagnosis. Data are mean (95% CI). BW, birthweight; LGA classified as BW >90th percentile for gestational age. ^a^This group may contain some patients with undiagnosed GDM due to fasting hyperglycaemia that is not identified by the GCT

Data were collected for demographic information, glucose screening results and pregnancy outcomes. Women were classified into groups according to their OGTT results: OGTT not done; NICE-negative IADPSG-negative; NICE-positive IADPSG-positive; NICE-negative IADPSG-positive; and NICE-positive IADPSG-negative (Table 1, Fig. 1). Groups were further divided according to the OGTT criterion that was abnormal. Women could test positive for some but not all GDM diagnostic criteria by having an FPG 5.1–5.5 mmol/l (IADPSG-only 0 h group), an OGTT 1 h glucose ≥10.0 mmol/l (IADPSG-only 1 h group) or an OGTT 2 h glucose 7.8–8.4 mmol/l (NICE-only 2 h group, which was also the NICE-positive IADPSG-negative group) (Table 1, Fig. 1).

Participant characteristics are presented as *n* (%) for categorical data and mean (95% CI) for continuous data. Differences between each OGTT classification group and the reference population were tested using Fisher’s exact test and linear regression, respectively. The reference population was considered to be all women who did not have an OGTT (*n* = 21,695). These women had a GCT result <7.8 mmol/l, but some might have had fasting hyperglycaemia that would not have been identified using the GCT.

Associations between GDM classification group and maternal or fetal outcomes compared with the reference population were estimated using logistic regression, and results are presented as ORs (95% CIs). Results are presented as unadjusted models and models which were adjusted for potential confounders [10]. Analyses of LGA, small for gestational age (SGA; birthweight <10th percentile for gestational age) and pre-eclampsia outcomes were adjusted for maternal BMI, maternal age, parity, maternal smoking and ethnicity. These analyses were not adjusted for gestational age at birth, as it was not considered a true confounder for pre-eclampsia, and LGA and SGA already incorporate gestational age within their definitions. Macrosomia, Caesarean section (CS), instrumental delivery, stillbirth, infant admission to the neonatal intensive care unit (NICU), polyhydramnios, ante- or postpartum haemorrhage, and 1 or 5 min Apgar scores were adjusted for maternal BMI, maternal age, parity, maternal smoking, ethnicity and estimated gestational age at birth. The preterm delivery outcome was adjusted for maternal BMI, maternal age, parity, maternal smoking, ethnicity, pre-eclampsia and antepartum haemorrhage. To make allowance for multiple testing, a significance level of *p* ≤ 0.001 was considered significant and *p* ≤ 0.01 was considered a trend. Statistical analysis was performed using Stata version 12.0 software (StataCorp LP, College Station, TX, USA).

## Results

Records were obtained for 25,789 births; 25,543 records were included in the analysis after exclusion of pregnancies resulting in miscarriage (*n* = 59) or termination (*n* = 65), those with no birthweight information (*n* = 3), duplicate data (*n* = 20) and records consistent with overt diabetes (RPG ≥11.1 mmol/l at booking; *n* = 99). Over 99.9% of records had data available for pregnancy outcome, mode of delivery and antenatal complications; 84.9% of records had data available for their usual maternal adult BMI. Characteristics of the study population are described in Table 2.

**Table 2:** Characteristics of pregnancies classified according to OGTT diagnosis

Characteristic	All pregnancies	OGTT not done	NICE-negative IADPSG-negative	NICE-positive IADPSG-positive	NICE-negative IAD-positive	NICE-positive IAD-negative (NICE-only 2 h)	IADPSG-only 0 h	IADPSG-only 1 h
*N*	25,543	21,695	2,406	794	387	261	167	288
Maternal age, years^a^	30.7 (30.6, 30.8)	30.5 (30.4, 30.6)	31.4 (31.2, 31.6)***	32.7 (32.4, 33.1)***	32.6 (32.1, 33.1)***	32.1 (31.5, 32.7)***	32.7 (31.9, 33.5)***	32.6 (32.0, 33.2)***
Maternal usual BMI, kg/m^2a^	24.7 (24.6, 24.8)	24.4 (24.3, 24.4)	26.0 (25.7, 26.2)***	27.6 (27.1, 28.1)***	27.4 (26.8, 28.1)***	25.5 (24.8, 26.2)***	29.0 (27.9, 30.1)***	27.2 (26.5, 27.9)***
Maternal ethnicity^c, d^				***	**			**
White	22,762 (89.3)	19,406 (89.4)	2,151 (89.4)	641 (80.7)	336 (86.8)	228 (87.4)	144 (86.2)	246 (85.4)
Black	423 (1.7)	376 (1.7)	28 (1.2)	10 (1.3)	4 (1.0)	5 (1.9)	1 (0.6)	3 (1.0)
Asian	1,353 (5.3)	1,077 (5.0)	126 (5.2)	92 (11.6)	36 (9.3)	22 (8.4)	18 (10.8)	31 (10.8)
Other	947 (3.7)	791 (3.6)	89 (3.7)	50 (6.3)	11 (2.8)	6 (2.3)	4 (2.4)	8 (2.8)
Maternal smoking^b^	2,416 (9.5)	2,150 (9.9)	177 (7.4)***	47 (5.9)***	27 (7.0)	15 (5.7)	11 (6.6)	21 (7.3)
Primiparous^b^	9,895 (38.7)	8,389 (38.7)	941 (39.1)	317 (39.9)	141 (36.4)	107 (41.0)	57 (34.1)	103 (35.8)
Estimated gestational age at delivery, weeks^a^	39.2 (39.1, 39.2)	39.2 (39.1, 39.2)	39.3 (39.3, 39.4)***	38.8 (38.6,38.9)***	39.1 (38.9, 39.2)	38.9 (38.7, 39.1)	39.1 (38.8, 39.4)	39.1 (38.8, 39.3)
Birthweight, g^a^	3,381.2 (3,373.8, 3,388.7)	3,360.7 (3,352.6, 3,368.8)	3,496.6 (3,473.9, 3,519.3)***	3,487.1 (3,446.2, 3,528.0)***	3,612.1 (3,551.1, 3,673.1)***	3,360.7 (3,294.7, 3,426.8)	3,711.1 (3,619.0, 3,803.2)***	3,574.2 (3,503.4, 3,645.0)***
Mode of delivery								
SVD	15,321 (60.0)	13,405 (61.8)	1,264 (52.5)	341 (42.9)	190 (49.1)	121 (46.4)	74 (44.3)	147 (51.0)
Emergency CS	3,937 (15.4)	3,132 (14.4)	473 (19.7)	190 (23.9)	94 (24.3)	48 (18.4)	44 (26.3)	68 (23.6)
Elective CS	2,858 (11.2)	2,254 (10.4)	342 (14.2)	157 (19.8)	53 (13.7)	52 (19.9)	29 (17.4)	35 (12.2)
Instrumental	3,319 (13.0)	2,802 (12.9)	324 (13.5)	104 (13.1)	50 (12.9)	39 (14.9)	20 (12.0)	38 (13.2)
Other/unknown	108 (0.4)	102 (0.5)	3 (0.1)	2 (0.3)	0 (0.0)	1 (0.4)	0 (0.0)	0 (0.0)
Complications								
LGA	3,010 (12.2)	2,264 (10.4)	406 (16.9)	195 (24.6)	115 (29.7)	30 (11.5)	63 (37.7)	75 (26.0)
Macrosomia	3,097 (12.1)	2,427 (11.2)	403 (16.8)	131 (16.5)	112 (28.9)	24 (9.2)	61 (36.5)	77 (26.7)
SGA	2,956 (12.0)	2,619 (12.5)	224 (9.6)	57 (7.3)	24 (6.4)	32 (12.6)	8 (4.9)	19 (6.8)
Stillbirth	85 (0.3)	77 (0.4)	5 (0.2)	2 (0.3)	1 (0.3)	0 (0.0)	0 (0.0)	1 (0.3)
Preterm	1,726 (6.8)	1,492 (6.9)	127 (5.3)	57 (7.2)	29 (7.5)	21 (8.0)	11 (6.6)	21 (7.3)
Pre-eclampsia	1,454 (5.7)	1,147 (5.3)	174 (7.2)	70 (8.8)	39 (10.1)	24 (9.2)	16 (9.6)	32 (11.1)
Polyhydramnios	283 (1.1)	122 (0.6)	106 (4.4)	32 (4.0)	15 (3.9)	8 (3.1)	5 (3.0)	13 (4.5)
APH	530 (2.1)	443 (2.0)	57 (2.4)	17 (2.1)	6 (1.6)	7 (2.7)	3 (1.8)	5 (1.7)
PPH	411 (1.6)	344 (1.6)	48 (2.0)	14 (1.8)	4 (1.0)	1 (0.4)	1 (0.6)	4 (1.4)
1 min Apgar score <7	1,537 (6.0)	1,297 (6.0)	141 (5.9)	50 (6.3)	34 (8.8)	15 (5.7)	18 (10.8)	25 (8.7)
5 min Apgar score <7	211 (0.8)	186 (0.9)	11 (0.5)	9 (1.1)	4 (1.0)	1 (0.4)	1 (0.6)	4 (1.4)
Infant admission to NICU	1,725 (6.8)	1,488 (6.9)	143 (5.9)	53 (6.7)	22 (5.7)	19 (7.3)	10 (6.0)	16 (5.6)
Treatment offered for GDM	1,017 (4.0)	0 (0.0)	0 (0.0)	756 (95.2)	0 (0.0)	261 (100.0)	0 (0.0)	0 (0.0)

Data are *n* (%) or mean (95% CI)

^a^Linear or ^b^logistic regression or ^c^Fisher’s exact test used to compare GDM classification groups with ‘OGTT not done’ group

^d^Ethnicity: significance testing indicates changes in ethnic composition between groups

**p ≤ 0.01; ***p ≤ 0.001

APH, antepartum haemorrhage; PPH, postpartum haemorrhage; SVD, spontaneous vertex delivery

A total of 3,848 (15.1%) antenatal OGTTs were performed, of which 2,406 (62.5%) were negative for GDM according to both IADPSG and the proposed NICE 2015 criteria, and 794 women (20.6%) had GDM according to both IADPSG and NICE 2015 criteria. In this study, the prevalence of GDM was 4.13% (1,055/25,543), using NICE 2015 criteria, and 4.62% (1,181/25,543) according to the IADPSG criteria. Using the IADPSG criteria instead of the proposed NICE 2015 criteria would have resulted in treating 126 more women over 5 years. Although these 126 women represented only 0.49% of pregnancies, they accounted for 3.82% of cases of LGA, 2.68% of cases of pre-eclampsia and 5.30% of cases of polyhydramnios. Overall, 3,010 (12.2%) infants had a birthweight above the 90th percentile, of whom 207 (6.9%) mothers had been offered treatment for hyperglycaemia.

### Characteristics of women with abnormal glucose tests

As expected, women with GDM diagnosed by any method were older and had a higher BMI compared with the general population (Table 2). Pregnancies complicated by one or more abnormal glucose values yielded an infant with a higher birthweight (Tables 2, 3). Women who were offered treatment for GDM delivered infants with an average birthweight of 3,437 g and a higher rate of macrosomia (adjusted OR 1.49 [1.21, 1.84]) and LGA (adjusted OR 1.84 [1.54, 2.20]) compared with the reference population, after adjustment for maternal age, parity, BMI, smoking status and ethnicity (and estimated gestational age at birth for macrosomia outcome). Women who had GDM by any criteria or both criteria were more likely to have a CS delivery and to suffer from pre-eclampsia compared with the reference population.

**Table 3:** Risk profiles of pregnancies classified according to OGTT diagnosis

Risk profile	OGTT not done	NICE-negative IADPSG-negative	NICE-positive IADPSG-positive	NICE-negative IADPSG-positive	NICE-positive IADPSG-negative (NICE-only 2 h)	IADPSG-only 0 h	IADPSG-only 1 h
*N*	21,695	2,406	794	387	261	167	288
Treatment offered	0 (0.0)	0 (0.0)	756 (95.2)	0 (0.0)	261 (100.0)	0 (0.0)	0 (0.0)
LGA	1.00 (Ref.)	1.75 (1.56, 1.96)***^a^ 1.63 (1.44, 1.84)***^b^	2.76 (2.34, 3.27)***^a^ 2.24 (1.86, 2.71)***^b^	3.64 (2.91, 4.56)***^a^ 3.12 (2.44, 3.98)***^b^	1.10 (0.75, 1.62)^a^ 1.10 (0.73, 1.65)^b^	5.24 (3.81, 7.21)***^a^ 4.47 (3.15, 6.33)***^b^	3.04 (2.33, 3.98)***^a^ 2.58 (1.93, 3.46)***^b^
Birthweight ≥4 kg	1.00	1.60 (1.42, 1.79)***^a^ 1.52 (1.34, 1.73)***^c^	1.57 (1.29, 1.90)***^a^ 1.87 (1.50, 2.33)***^c^	3.23 (2.59, 4.04)***^a^ 3.55 (2.75, 4.58)***^c^	0.80 (0.53, 1.23)^a^ 0.97 (0.62, 1.52)^c^	4.57 (3.33, 6.28)***^a^ 5.02 (3.46, 7.28)***^c^	2.90 (2.22, 3.77)***^a^ 3.21 (2.38, 4.34)***^c^
SGA	1.00	0.75 (0.65, 0.86)***^a^ 0.80 (0.68, 0.94)**^b^	0.55 (0.42, 0.73)***^a^ 0.62 (0.46, 0.84)**^b^	0.48 (0.32, 0.72)***^a^ 0.50 (0.32, 0.79)**^b^	1.01 (0.69, 1.46)^a^ 0.90 (0.59, 1.39)^b^	0.36 (0.18, 0.74)**^a^ 0.36 (0.16, 0.83)^b^	0.51 (0.32, 0.82)**^a^ 0.57 (0.35, 0.94)^b^
Emergency CS	1.00	1.45 (1.30, 1.61)***^a^ 1.31 (1.16, 1.47)***^c^	1.86 (1.58, 2.20)***^a^ 1.38 (1.14, 1.67)***^c^	1.90 (1.50, 2.41)***^a^ 1.60 (1.24, 2.06)***^c^	1.34 (0.98, 1.84)^a^ 1.12 (0.80, 1.58)^c^	2.12 (1.50, 3.00)***^a^ 1.66 (1.13, 2.43)**^c^	1.81 (1.39, 2.41)***^a^ 1.49 (1.10, 2.01)**^c^
All CS	1.00	1.55 (1.42, 1.70)***^a^ 1.36 (1.23, 1.51)***^c^	2.35 (2.04, 2.71)***^a^ 1.59 (1.35, 1.87)***^c^	1.85 (1.51, 2.28)***^a^ 1.44 (1.15, 1.81)**^c^	1.89 (1.47, 2.43)***^a^ 1.53 (1.16, 2.02)**^c^	2.35 (1.73, 3.20)***^a^ 1.71 (1.22, 2.39)**^c^	1.69 (1.32, 2.15)***^a^ 1.29 (0.99, 1.68)^c^
Instrumental delivery	1.00	1.05 (0.93, 1.19)^a^ 1.06 (0.93, 1.21)^c^	1.02 (0.82, 1.25)^a^ 1.15 (0.91, 1.46)^c^	1.00 (0.74, 1.35)^a^ 1.00 (0.72, 1.40)^c^	1.19 (0.84, 1.68)^a^ 1.18 (0.82, 1.72)^c^	0.92 (0.57, 1.47)^a^ 0.94 (0.55, 1.61)^c^	1.02 (0.73, 1.44)^a^ 1.03 (0.70, 1.52)^c^
Stillbirth	1.00	0.58 (0.24, 1.45)^a^ 1.09 (0.42, 2.79)^c^	0.71 (0.17, 2.89)^a^ 1.00 (0.23, 4.24)^c^	0.73 (0.10, 5.24)^a^ 1.16 (0.16, 8.70)^c^	Insufficient events	Insufficient events	0.98 (0.14, 7.06)^a^ 1.46 (0.19, 11.09)^c^
Infant admitted to NICU	1.00	0.86 (0.72, 1.02)^a^ 1.09 (0.89, 1.35)^c^	0.97 (0.73, 1.29)^a^ 0.83 (0.59, 1.16)^c^	0.82 (0.53, 1.26)^a^ 0.76 (0.45, 1.29)^c^	1.06 (0.66, 1.70)^a^ 1.06 (0.61, 1.85)^c^	0.86 (0.45, 1.64)^a^ 1.05 (0.51, 2.14)^c^	0.80 (0.48, 1.33)^a^ 0.65 (0.34, 1.23)^c^
Preterm delivery	1.00	0.75 (0.63, 0.91)^a^ 0.72 (0.59, 0.89)**^d^	1.05 (0.80, 1.38)^a^ 1.05 (0.78, 1.41)^d^	1.10 (0.75, 1.61)^a^ 1.02 (0.68, 1.55)^d^	1.18 (0.76, 1.86)^a^ 1.06 (0.64, 1.74)^d^	0.95 (0.52, 1.76)^a^ 0.88 (0.46, 1.71)^d^	1.06 (0.68, 1.67)^a^ 0.97 (0.60, 1.57)^d^
Pre-eclampsia	1.00	1.40 (1.18, 1.65)***^a^ 1.21 (1.01, 1.44)^b^	1.73 (1.35, 2.23)***^a^ 1.17 (0.88, 1.55)^b^	2.01 (1.43, 2.81)***^a^ 1.40 (0.97, 2.03)^b^	1.81 (1.19, 2.77)**^a^ 1.58 (1.01, 2.47)^b^	1.90 (1.13, 3.19)^a^ 1.12 (0.63, 1.99)^b^	2.24 (1.54, 3.25)***^a^ 1.66 (1.11, 2.48)^b^
Polyhydramnios	1.00	8.15 (6.26, 10.61)***^a^ 7.90 (5.94, 10.53)***^c^	7.43 (5.00, 11.03)***^a^ 6.94 (4.55, 10.58)***^c^	7.13 (4.13, 12.31)***^a^ 6.90 (3.94, 12.08)***^c^	5.59 (2.71, 11.56)***^a^ 6.13 (2.93, 12.76)***^c^	5.46 (2.20, 13.53)***^a^ 4.67 (1.83, 11.89)***^c^	8.36 (4.66, 14.99)***^a^ 7.46 (4.06, 13.72)***^c^
APH	1.00	1.16 (0.88, 1.54)^a^ 1.31 (0.97, 1.77)^c^	1.05 (0.64, 1.71)^a^ 0.95 (0.54, 1.67)^c^	0.76 (0.34, 1.70)^a^ 0.60 (0.22, 1.62)^c^	1.32 (0.62, 2.82)^a^ 1.59 (0.74, 3.40)^c^	0.88 (0.28, 2.76)^a^ 1.06 (0.33, 3.36)^c^	0.85 (0.35, 2.06)^a^ 0.59 (0.19, 1.85)^c^
PPH	1.00	1.26 (0.93, 1.71)^a^ 1.36 (0.99, 1.88)^c^	1.11 (0.65, 1.91)^a^ 0.92 (0.50, 1.71)^c^	0.65 (0.24, 1.75)^a^ 0.69 (0.25, 1.86)^c^	0.24 (0.03, 1.71)^a^ 0.26 (0.04, 1.86)^c^	0.37 (0.05, 2.68)^a^ 0.39 (0.05, 2.83)^c^	0.87 (0.32, 2.36)^a^ 0.93 (0.34, 2.52)^c^
1 min Apgar score <7	1.00	0.98 (0.82, 1.17)^a^ 1.05 (0.86, 1.28)^c^	1.05 (0.79, 1.41)^a^ 0.87 (0.62, 1.21)^c^	1.51 (1.06, 2.16)^a^ 1.55 (1.06, 2.26)^c^	0.96 (0.57, 1.61)^a^ 0.93 (0.53, 1.65)^c^	1.88 (1.15, 3.08)^a^ 2.16 (1.30, 3.60)**^c^	1.49 (0.99, 2.26)^a^ 1.48 (0.94, 2.31)^c^
5 min Apgar score <7	1.00	0.53 (0.29, 0.97)^a^ 0.76 (0.40, 1.47)^c^	1.32 (0.67, 2.59)^a^ 1.32 (0.60, 2.91)^c^	1.21 (0.45, 3.27)^a^ 1.36 (0.43, 4.38)^c^	0.44 (0.06, 3.16)^a^ 0.68 (0.09, 4.96)^c^	0.69 (0.10, 4.98)^a^ 1.03 (0.14, 7.53)^c^	1.63 (0.60, 4.42)^a^ 1.88 (0.58, 6.05)^c^

Data are *n* (%) or OR (95% CI)

^a^Unadjusted

^b^After adjustment for maternal BMI, maternal age, parity, maternal smoking and ethnicity

^c^After adjustment for maternal BMI, maternal age, parity, maternal smoking, ethnicity and estimated gestational age at birth

^d^After adjustment for maternal BMI, maternal age, parity, maternal smoking, ethnicity, pre-eclampsia and antepartum haemorrhage

** *p* ≤ 0.01

***p ≤ 0.001

APH, antepartum haemorrhage; PPH, postpartum haemorrhage; Ref., reference; SVD, spontaneous vertex delivery

Pregnancies where an OGTT was performed that was negative for GDM according to the NICE and IADPSG criteria (NICE-negative IADPSG-negative) were at higher risk of macrosomia (16.8%; unadjusted OR 1.60 [1.42, 1.79], adjusted OR 1.52 [1.34, 1.73]), LGA (16.9%; unadjusted OR 1.75 [1.56, 1.96], adjusted OR 1.63 [1.44, 1.84]), CS delivery (33.9%; unadjusted OR 1.55 [1.42, 1.70], adjusted OR 1.36 [1.23, 1.51]) (especially emergency CS [19.7%; unadjusted OR 1.45 (1.30, 1.61), adjusted OR 1.31 (1.16, 1.47)]), pre-eclampsia (7.2%; unadjusted OR 1.40 [1.18, 1.65], adjusted model did not show a significant effect) and polyhydramnios (4.4%; unadjusted OR 8.15 [6.26, 10.61], adjusted OR 7.90 [5.94, 10.53]) compared with the reference population. These women had abnormal glucose tests on screening and/or a history of previous GDM.

Women who were NICE-positive IADPSG-negative on OGTT were offered treatment and had an LGA rate comparable to that of the reference population (adjusted OR 1.10 [0.73, 1.65]; 11.5% vs 10.4%; Table 3). Overall, there was a trend for increased CS rates in this group (adjusted OR 1.53 [1.16, 2.02; *p* = 0.003]), but there was no increase in emergency CS rates (adjusted OR 1.12 [0.80, 1.58]). Pregnancies in this category were at increased risk of polyhydramnios (adjusted OR 6.13 [2.93, 12.76]) but not of pre-eclampsia (adjusted OR 1.58 [1.01, 2.47]) compared with the reference population.

Interestingly, women in the NICE-negative IADPSG-positive category who were untreated had the highest rate of LGA in this study (29.7%; adjusted OR 3.12 [2.44, 3.98]). These women were at higher risk of emergency CS delivery (adjusted OR 1.60 [1.24, 2.06]) and polyhydramnios (adjusted OR 6.90 [3.94, 12.08]) compared with the reference population.

### Women who fall through the net with NICE 2015: identifying the group at highest risk of LGA and other adverse outcomes

Women in both the IADPSG-only 0 h and IADPSG-only 1 h groups were at high risk of LGA, CS delivery and emergency CS compared with the reference population, but the risks were higher for the IADPSG-only 0 h group: 37.7% and 26.0% of IADPSG-only 0 h and IADPSG-only 1 h pregnancies, respectively, yielded an LGA infant. Infants in the IADPSG-only 0 h group had a mean birthweight of 3,711.1 g: 350.4 g higher than infants from the reference population. They also had a trend for an increased risk of having a low 1 min Apgar score (adjusted OR 2.16 [1.30, 3.60]; *p* = 0.003). Pregnancies in the IADPSG-only 1 h group had the greatest risk of polyhydramnios (adjusted OR 7.46 [4.06, 13.72]) and pre-eclampsia (unadjusted OR 2.24 [1.54, 3.25]) when compared with the reference population. However, when adjustment was made for maternal BMI, age, parity, smoking, ethnicity and estimated gestational age at birth, the risk of pre-eclampsia was no longer significant.

Overall, 20.4% of women who were offered treatment for hyperglycaemia in pregnancy had an LGA infant. Although many of these women had more severe hyperglycaemia, this information was used to give a conservative estimate for the number needed to treat (NNT) to prevent one case of LGA in the groups which did not receive treatment. NNTs for the IADPSG-only 0 h and IADPSG-only 1 h groups were 5.8 and 17.9, respectively.

## Discussion

In this study, the prevalence of GDM in the study population was 4.13% using NICE 2015 criteria and 4.62% using IADPSG criteria. Using the IADPSG criteria instead of the proposed NICE 2015 criteria would have resulted in treating 126 more women over 5 years. Using the proposed NICE 2015 criteria would have resulted in 0.49% fewer women being offered treatment but would have failed to identify 3.82% cases of LGA, 2.68% cases of pre-eclampsia and 5.30% cases of polyhydramnios.

In this study, the women at highest risk of having an LGA infant were those who had an abnormal OGTT but ‘fell through the net’ between the IADPSG and NICE 2015 diagnostic cut-offs for GDM. Many of these women were not identified according to the diagnostic thresholds used at the time in our institution (75 g OGTT 0 h ≥6.1 mmol/l or ≥7.1 mmol/l; 2 h ≥7.8 mmol/l) and therefore were not offered treatment. Women with more severe degrees of hyperglycaemia were offered treatment and their offspring had a much lower risk of being LGA. Previous reports have suggested that offering treatment to women with modest degrees of hyperglycaemia in pregnancy results in a reduction in birthweight of 100–140 g [13]. However, in this study, women with an FPG 5.1–5.5 mmol/l (IADPSG-only 0 h) gave birth to infants with a very high rate of LGA (37.7%) and a mean birthweight 350 g higher than that of the reference population and 274 g higher than that of women with treated hyperglycaemia. A detailed cost–benefit analysis was beyond the scope of this project, but an NNT of 5.8 for this group suggests that treatment may be economical depending upon the expected risks in the population and compares favourably to other interventions in diabetes [22].

This study has several strengths. Data were collected on all singleton pregnancies at our institution between 2004 and 2008. This allows the assessment of outcomes and risk in a real-life clinical setting. Unlike many prospective clinical studies, the individuals were not highly selected and there was no difference between their care and standard clinical care at the time. However, this was a single centre study in a population with relatively low levels of ethnic diversity. Overall, the prevalence of GDM in our population was relatively low. Higher incidence rates for hyperglycaemia in pregnancy have been reported in other regions worldwide [23]. The IADPSG guidelines [11] now recommend screening pregnant women who are not known to have diabetes at 24–28 weeks’ gestation using the OGTT and not the GCT. The use of the OGTT for universal screening of pregnant women may be associated with an increase in the prevalence of GDM in our population. However, the increased use of the OGTT also makes the issue of ‘falling through the net’ more important, as the number of women who test positive for the IADPSG and negative for the NICE 2015 criteria will be higher.

Using a retrospective study design confers some disadvantages. First, the screening protocol in use at the time used a GCT to exclude women who did not need an OGTT. Women with fasting hyperglycaemia, shown in this study to be at risk of adverse outcomes, would not have been readily identified using a GCT, which relies on a 1 h post-load test only. However, if these women had been removed from the analysis, the reference population would have been lower risk overall and the differences in outcome between reference and study populations would have been similar or more marked. Although screening tests were offered to all pregnant women, a minority might have chosen not to be screened, or not to attend for an OGTT if an initial screening test was positive. All women meeting predetermined criteria were offered dietary advice and other treatment, but response to treatment offered, the nature and dose of such treatments, and adherence to any advice or treatments received are unclear. Women with GDM received more antenatal scans and appointments and may be at greater risk of interventions or conditions such as pre-eclampsia and polyhydramnios through increased contact with health professionals. Although we cannot adequately control for this, it is interesting to note that women who were NICE-negative IADPSG-positive also had increased rates of pre-eclampsia and CS despite being considered not to have GDM at the time. Some capillary blood glucose samples were used in place of venous plasma for blood glucose measurement, which might have introduced small variations in measured glucose concentrations. A further consideration is that following an abnormal GCT result, some women might have instituted lifestyle change prior to OGTT testing, producing a better-than-expected OGTT result. While lifestyle change has been shown to improve glucose tolerance in obese pregnant women [24], the 1–2 week window between an abnormal GCT and a follow-up OGTT gives limited time for this, and further opportunities for GDM diagnosis were available later in pregnancy for women with symptoms or ultrasound features consistent with macrosomia. Information on neonatal sex was unavailable and therefore sex-specific criteria for LGA could not be used [25]. Although the sample size was large overall, the number of women in certain subgroups was too small to enable meaningful analysis of rare outcomes such as stillbirth. A 75 g OGTT was used, rather than the 100 g OGTT favoured by the American College of Obstetricians and Gynecologists (ACOG), which does not permit direct comparisons to be made with the ACOG diagnostic criteria. However, the ACOG criteria (100 g OGTT 0 h ≥5.3 mmol/l; 1 h ≥10.0 mmol/l; 2 h ≥8.6 mmol/l; 3 h ≥7.8 mmol/l) will also identify many women with fasting hyperglycaemia, although as two abnormal results are required to make a diagnosis not all in this group would be offered treatment.

There is a fundamental difference between the IADPSG and NICE 2015 criteria. The IADPSG criteria are grounded on minimising the risk of harm to the mother and baby, and diagnostic thresholds were set to give an OR of 1.75 at each OGTT time point [11]. One of the challenges in ascertaining GDM-related risks in pregnancy is the large number of relatively infrequent adverse outcomes that have not shown consistent evidence of a significant association with GDM. For example, in the current study of 25,543 pregnancies, there were insufficient events to determine any effect of GDM classification on stillbirth rates or maternal admission to intensive care, and there was no evidence of altered rates of infant admission to the NICU. However, despite these concerns, the IADPSG criteria have been shown to be cost-effective, at least partly because of a reduction in the risk of infrequent adverse effects such as NICU admission [26]. Conversely, the NICE 2015 criteria have been based upon reducing historical average National Health Service unit costs for selected adverse outcomes (those common enough to have statistical power to be detected in randomised controlled studies) using health economic modelling. While cost-effectiveness is important in any healthcare system, the burden of psychological and emotional distress caused by many complications is also important and cannot be measured in economic terms alone. GDM diagnostic criteria should aim to identify women at risk of complications in the first instance, although not all these women may require pharmacological treatment. Future work should focus on accurate identification of women with GDM who are at low risk of complications and who might be suitable for community-based lifestyle interventions.

This study demonstrates a significant risk of LGA among infants of women who meet the IADPSG criteria for GDM who would be unidentified and untreated using many criteria in current clinical use (WHO 1999, modified WHO 1999 and proposed NICE 2015) [27]. One barrier to adoption of the IADPSG criteria for GDM has been the widespread concern about increased case identification rates and increased treatment rates leading to increased costs with purportedly little outcome data to support such a change [12]. Some reports have suggested that diagnosis rates of GDM would increase dramatically under the IADPSG criteria [13]. The current dataset suggests that adopting the IADPSG criteria over the NICE 2015 guidelines would be associated with a small increase in the prevalence of GDM from 4.13% to 4.62% in this population if screening protocols were unchanged (an additional 25.2 patients per year). Worryingly, these data suggest that if the NICE 2015 criteria were adopted instead of the IADPSG criteria a group of high-risk women would be unidentified and undertreated (IADPSG-only 0 h) and a group of low-risk women (NICE-only 2 h) would be treated instead. This suggests that despite being based upon cost-effectiveness, the NICE criteria may not necessarily facilitate the allocation of resources to address those most at risk of adverse outcomes.

The IADPSG criteria include women with an FPG 5.1–5.5 mmol/l and an OGTT 1 h glucose ≥10.0 mmol/l who, untreated, were associated with a high risk of delivering LGA infants in this study. The IADPSG criteria exclude women who have an OGTT 2 h glucose 7.8–8.4 mmol/l, who were found in this study to have a very low risk of LGA in their offspring (11.5%). Although many in this group would have been offered treatment, a confounding factor, the low rate of LGA, suggests that some of these women could be safely untreated. Interestingly, these treated women had a lower incidence of LGA among their infants compared with untreated women who had an OGTT that was negative for GDM according to both NICE and IADPSG criteria despite having abnormal screening tests or previous GDM. The offspring of these women with a negative OGTT had a 16.9% risk of LGA, significantly higher than that of the reference population. These results confirm that women with borderline hyperglycaemia who have positive screening tests but who do not meet the diagnostic thresholds for GDM are also at increased risk of adverse outcomes. This finding suggests that standard dietary and lifestyle advice given in GDM might benefit even non-GDM women in pregnancy and could be given more widely.

One issue which has prevented widespread adoption of the IADPSG diagnostic criteria is the concern about unnecessarily over-medicalising healthy pregnancies [13]. In this study, even women with a negative OGTT were at higher risk of CS delivery compared with the reference population. The highest risks of CS delivery in this study were seen in women with a positive OGTT (NICE-positive IADPSG-positive) and in women with an FPG 5.1–5.5 mmol/l (IADPSG-only 0 h). Recommendations to offer induction at 38 weeks to women with treated GDM are likely to explain the increased rates of emergency CS in the NICE-positive IADPSG-positive group due to the risk of failed induction [17]. However, the IADPSG-only 0 h group had higher rates of emergency CS delivery overall, suggesting that women with unidentified hyperglycaemia had increased intervention rates despite being considered GDM-negative according to the diagnostic criteria in use in our institution at the time. These findings suggest that intervention rates may be related to glucose per se rather than diagnostic categorisation using the OGTT.

In summary, women who fall through the net, who would test positive for GDM according to the IADPSG criteria but not the NICE 2015 criteria, had the highest risk of having infants with LGA in this retrospective study compared with women in the reference population or those with more severe degrees of hyperglycaemia who were offered treatment. Women with FPG levels 5.1–5.5 mmol/l were at particularly high risk of CS delivery and LGA. Conversely, women with an OGTT 2 h glucose 7.8–8.4 mmol/l, who would have had GDM according to the NICE 2015 criteria but not the IADPSG criteria, were offered treatment and had an extremely low incidence of LGA. These data demonstrate that the IADPSG criteria identify women at substantial risk of LGA who may benefit from treatment while excluding women who may have a low risk of adverse pregnancy outcomes.

## Abbreviations

ACOG: American College of Obstetricians and Gynecologists
CS: Caesarean section
FPG: Fasting plasma glucose
GDM: Gestational diabetes mellitus
GCT: Glucose challenge test
IADPSG: International Association of the Diabetes and Pregnancy Study Groups
LGA: Large for gestational age
NICE: National Institute for Health and Care Excellence
NICU: Neonatal intensive care unit
NNT: Number needed to treat
RPG: Random plasma glucose
SGA: Small for gestational age
